# Hypoxic Repeat Sprint Training Improves Rugby Player's Repeated Sprint but Not Endurance Performance

**DOI:** 10.3389/fphys.2017.00024

**Published:** 2017-02-07

**Authors:** Michael J. Hamlin, Peter D. Olsen, Helen C. Marshall, Catherine A. Lizamore, Catherine A. Elliot

**Affiliations:** ^1^Department of Tourism, Sport and Society, Lincoln UniversityChristchurch, New Zealand; ^2^Department of Nursing, Midwifery and Allied Health, Ara Institute of CanterburyChristchurch, New Zealand

**Keywords:** normobaric hypoxia, Yo-Yo intermittent recovery test, team sports, repeated sprint ability, intermittent hypoxic training

## Abstract

This study aims to investigate the performance changes in 19 well-trained male rugby players after repeat-sprint training (six sessions of four sets of 5 × 5 s sprints with 25 s and 5 min of active recovery between reps and sets, respectively) in either normobaric hypoxia (HYP; *n* = 9; F_I_O_2_ = 14.5%) or normobaric normoxia (NORM; *n* = 10; F_I_O_2_ = 20.9%). Three weeks after the intervention, 2 additional repeat-sprint training sessions in hypoxia (F_I_O_2_ = 14.5%) was investigated in both groups to gauge the efficacy of using “top-up” sessions for previously hypoxic-trained subjects and whether a small hypoxic dose would be beneficial for the previously normoxic-trained group. Repeated sprint (8 × 20 m) and Yo-Yo Intermittent Recovery Level 1 (YYIR1) performances were tested twice at baseline (Pre 1 and Pre 2) and weekly after (Post 1–3) the initial intervention (intervention 1) and again weekly after the second “top-up” intervention (Post 4–5). After each training set, heart rate, oxygen saturation, and rate of perceived exertion were recorded. Compared to baseline (mean of Pre 1 and Pre 2), both the hypoxic and normoxic groups similarly lowered fatigue over the 8 sprints 1 week after the intervention (Post 1: −1.8 ± 1.6%, −1.5 ± 1.4%, mean change ± 90% CI in HYP and NORM groups, respectively). However, from Post 2 onwards, only the hypoxic group maintained the performance improvement compared to baseline (Post 2: −2.1 ± 1.8%, Post 3: −2.3 ± 1.7%, Post 4: −1.9 ± 1.8%, and Post 5: −1.2 ± 1.7%). Compared to the normoxic group, the hypoxic group was likely to have substantially less fatigue at Post 3–5 (−2.0 ± 2.4%, −2.2 ± 2.4%, −1.6 ± 2.4% Post 3, Post 4, Post 5, respectively). YYIR1 performances improved throughout the recovery period in both groups (13–37% compared to baseline) with unclear differences found between groups. The addition of two sessions of “top-up” training after intervention 1, had little effect on either group. Repeat-sprint training in hypoxia for six sessions increases repeat sprint ability but not YYIR1 performance in well-trained rugby players.

## Introduction

Rugby union is a fast-paced, field-based sport where strength, power, speed, and endurance are essential (Nicholas, 1997). Moreover, because of the game's intermittent nature, the ability to sprint repetitively is also an important fitness component for the modern rugby player and may be crucial to the outcome of the game (Austin et al., 2011a). In team sports, although different playing positions require different anthropometric and physiological characteristics, the ability to perform repeated sprints is associated with improved measures of game performance (Rampinini et al., 2007). Traditionally, repeat-sprint training involved on-feet, repeated running bouts interspersed with appropriate recovery periods (Tønnessen et al., 2011). Such training has been shown to improve oxygen utilization (Bailey et al., 2009) and increase anaerobic metabolism (Dawson et al., 1998), thereby enhancing repeat sprint ability. However, because the aerobic system is heavily involved in regenerating ATP during recovery from repeated sprints (Spencer et al., 2005), it is thought that strategies used to improve aerobic metabolism may also help to improve repeat sprint ability. As a consequence, there has been an increased interest in the ability of altitude or hypoxic training to enhance repeat sprint ability.

Increased anaerobic glycolytic activity (Svedenhag et al., 1991 #2736; Faiss et al., 2013b) and modified acid-base homeostasis (Nummela and Rusko, 2000) have both been signaled as possible mechanisms responsible for the improved repeat sprint ability after altitude/hypoxic training. A lower rate of oxygen delivery to the muscle during hypoxic training probably increases the stress on the anaerobic metabolic pathways thereby resulting in upregulation of anaerobic metabolism (Faiss et al., 2013b).

However, adding hypoxia to repeat-sprint training has not always resulted in improved repeat sprint ability at sea-level. Faiss et al. (2013b) found that two repeat-sprint training sessions per week (3 sets of 5 × 10 s all-out cycle sprinting at ~3000 m) for 4 weeks had little effect on overall power output during a repeat sprint cycling test. Nevertheless, such training increased the number of all-out 10 s cycling sprints able to be completed prior to exhaustion in the hypoxic (3.6) compared to the normoxic (−0.4) trained groups (Faiss et al., 2013b). On the other hand, Goods et al. (2015) found 15 sessions of repeat-sprint training in hypoxia over 5 weeks (3 sets of 7 × 5 s all-out cycle sprints at ~3000 m), had little effect compared to similar exercise in normoxia on running or cycling repeat sprint ability in Australian football players. While Galvin et al. (2013) found repeat-sprint training (12 sessions over 4 weeks of 1 set of 10 × 6 s all-out treadmill sprints at ~3500 m) in hypoxic compared to normoxic conditions had little effect on repeat sprint ability in trained rugby players. Other researchers have reported adding hypoxia to repeat-sprint training can produce trivial (~1.5%) (Brocherie et al., 2015a) to substantial and long-lasting beneficial effects (2.9% immediately and 2.8% 3-weeks post-training) on repeat sprint ability (Brocherie et al., 2015b). In female participants, conflicting results also exist with some researchers reporting beneficial improvements in repeat sprint ability in the hypoxic compared to the normoxic trained group (Kasai et al., 2015), while others found no such change in repeat sprint ability (Jones B. et al., 2015). For a more detailed review of the use of repeat-sprint training in hypoxia see (Faiss et al., 2013a).

Repeat-sprint training under hypoxic conditions to improve endurance performance has also resulted in conflicting results. Galvin et al. (2013) found significantly improved endurance performance (Yo-Yo intermittent recovery test level 1) after repeat-sprint training in rugby players. More recently, Jones B. et al. (2015) reported significantly faster final running velocities in female field hockey players during a 30–15 intermittent field test after repeat-sprint training under hypoxic compared to control conditions, whereas others have found little effect on 20 m shuttle run performance post hypoxic repeat-sprint training (Goods et al., 2015).

Some of the variation in response to repeat-sprint training under hypoxia between studies is probably due to the methodological differences. While most studies use a similar hypoxic training stimulus (F_I_O_2_ ~14.5% equivalent to ~3000 m), the repeat-sprint training protocols can vary considerably from repetitions of short duration high-intensity repeats (12 sessions of 1 set of 10 × 6 s; Galvin et al., 2013), to longer-duration and subsequently lower-intensity repeats (six sessions of one set of between eight and 12 × 60 s; Jones M. R. et al., 2015). Exercise prescription theory dictates that exercise training prescription should be as specific as possible to facilitate appropriate physiological adaptations (Reilly et al., 2009). Therefore, repeat-sprint training protocols should be based on work-to-rest ratio's of actual repeat sprint ability while playing the sport (adding hypoxia to this training serves to induce a larger metabolic stimulus resulting in greater adaptation). Using GPS technology (MinimaxX, Catapult Innovation, 10 Hz) Jones M. R. et al. (2015) reported the repeat sprint efforts from 33 professional rugby players over the 2012-2013 season. On average, there were ~8 repeated high-intensity bouts (range from 5 to 11) per game, which had 3–4 efforts per bout with ~5 s between efforts and about 6–12 min recovery between bouts (Jones M. R. et al., 2015). Using time-motion analysis of 20 professional rugby players during the 2008-2009 Super 14 international rugby competition (Austin et al., 2011b), others have reported slightly more bouts of repeat sprint efforts during the game (mean 14, range 7–17), but a similar average recovery between bouts of ~6 min. The authors did not include the rest between efforts in each bout, so comparing the average duration of the bout (~30 s) is difficult. To our knowledge, there are few research studies that have based their repeat-sprint training protocols on data from real matches, particularly when adding hypoxia to the training. Therefore, the aim of this study was to examine the effect of using rugby-specific repeat-sprint training with hypoxia on field-based repeat sprint and endurance ability in rugby players during pre-season training.

It has been suggested that after traditional altitude training there is a small period of attenuated performance (due to the re-establishment of sea-level training volume and intensity), followed by a longer period of improved performance (Millet et al., 2010), as adaptations to the altitude training continue to manifest over time. While the post-training performance period requires further research, we have found that anaerobic performance in well-trained athletes started to decline back to baseline levels 9 days post-intermittent hypoxic training (Hamlin et al., 2010). Because of the natural degradation of the hypoxic adaptations over time, some practitioners have suggested using “top-up” sessions (hypoxic sessions of shorter duration than the initial hypoxic training spaced through-out the training year) in an attempt to maintain beneficial adaptations and performance (Saunders et al., 2009). The effect of a hypoxic repeat-sprint training top-up session on subsequent repeat sprint ability has not been researched to date.

Little information also exists on the minimum number of hypoxic exposures required to improve performance, or how long a possible hypoxic-induced performance enhancement remains. Therefore, a secondary aim of this study was to measure performance 3 weeks post-intervention to gain insight into the longevity of performance change, but also to investigate the effect of a 1-week top-up dose on previously hypoxic and normoxic-trained subjects.

## Materials and methods

### Subjects

Nineteen representative and club rugby players from Canterbury, New Zealand participated in this study. Players were non-professional and played in the senior and under 21 age-group teams in the Canterbury country competition. These players typically complete two rugby-specific and 1–2 strength and conditioning sessions per week during the pre-season and two rugby-specific, one game and one recovery session per week during the regular season. The research was conducted over the whole pre-season training period which typically lasts 8–10 weeks. This study was carried out in accordance with the recommendations of the Lincoln University Human Ethics guidelines with written informed consent from all subjects. All subjects gave their written informed consent in accordance with the Declaration of Helsinki. The protocol was approved by the Lincoln University Human Ethics Committee (reference 2015-46). Participant characteristics are presented in Table 1. All participants were healthy, free from injury, lived at sea level and had not resided at altitude within the previous 6 months. Participants were matched for baseline repeat sprint ability (i.e., cumulated time required to complete 8 × 20 m sprints), and then randomly divided into two groups: a hypoxic group (HYP, *n* = 9) and a control group (NORM, *n* = 10). Participants were asked to maintain their usual pre-season fitness and rugby training sessions throughout the study. Due to injury, one control group participant had to withdraw from the study.

**Table 1:** **Characteristics and baseline test 1 performance measures**.

	**NORM (*n* = 10)**	**HYP (*n* = 8)**
Age (yr)	22.0 ± 4.1	20.3 ± 2.1
Body mass (kg)	88.3 ± 14.1	77.1 ± 10.2^*^
Height (cm)	177.9 ± 5.4	173.9 ± 4.9
Weekly training (min.wk^−1^)	248.2 ± 208.9	270.9 ± 155.8
Weekly Trimp	3463 ± 3187	3642 ± 2191
Cumulated sprint time (s)	27.4 ± 3.2	26.9 ± 3.4
Repeated sprint fatigue^1^ (%)	5.5 ± 2.3	5.8 ± 3.4
Repeated sprint fatigue^2^ (%)	3.5 ± 1.2	3.5 ± 1.3
Yo-Yo level 1 (m)	1100 ± 426	1200 ± 384

Data are raw means ± SD. Weekly Trimp, weekly training impulse (training duration × intensity); Cumulated sprint time, total time for 8 sprints; Repeated sprint fatigue^1^, % fatigue from sprint 1 to sprint 8 using the linear extrapolation method; Repeated sprint fatigue^2^, % fatigue decrement score using the % decrement score method (100 × total sprint time/ideal sprint time) − 100; Yo-Yo level 1; distance covered in the Yo-Yo level 1 intermittent recovery test

* *Substantial differences*.

### Study design

**Figure 1:**
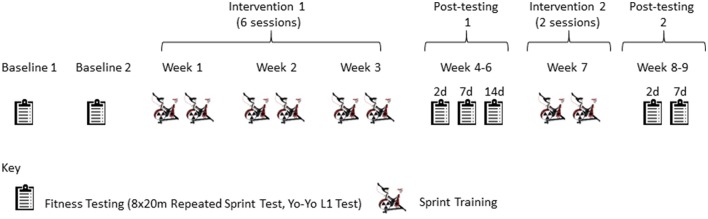
**Outline of training and testing schedule**.

This study was single blind, placebo-controlled trial whose intervention was based on repeat sprint bouts found in real rugby matches (Austin et al., 2011b; Jones M. R. et al., 2015). Participants performed seven main trials including two baseline and five post-exposure trials. The baseline trials were performed 4–5 days apart and 1 week before beginning the first repeat-sprint training block. After 3 weeks of repeat-sprint training, three post-training trials were completed (1 week apart). A second 1-week repeat-sprint training block followed where both groups trained under hypoxia. Two further post-training trials (1-week apart) followed the “top-up” sessions (see Figure 1). The main trials involved field-based fitness tests including a repeat sprint ability test (8 × 20 m all-out running sprints timed to go every 20 s) and a Yo-Yo Intermittent Recovery Level 1 test (YYIR1) commonly used on rugby players (Gore, 2000). To aid in the blinding, all participants were under the impression that they would be receiving altitude training (i.e., breathing hypoxic air).

### Participant preparation

The participants were asked to arrive in a fully rested and hydrated state and to refrain from intense exercise for 24 h and caffeine for 12 h prior to each main trial. All testing was performed at the same time of day (±1 h) to minimize diurnal variation. Participants were also asked to record their dietary intake before the first baseline fitness trial to allow for replication of the diet prior to subsequent trials.

### Hypoxic and normoxic repetitive sprint training

Participants were asked to complete repeat-sprint training on a Wattbike (Wattbike Pro, Nottingham, UK) at air brake resistance level 3, magnetic setting three for training sessions 1–2 and 5–8. The settings were increased for training sessions 3 and 4 (air brake level 5, magnetic setting 3) to increase overload and further challenge the participants. Prior to training, the zero was calibrated for each Wattbike according to the manufacturer's recommendations. Bike dimensions for individual athletes (saddle, handlebar heights, and positions) were initially recorded and replicated at each training session. Participants were asked to cycle in an upright, seated position. Training consisted of six sessions of repeat-sprint intervals over an initial 3 week period (two sessions per week), followed 3 weeks later by a further two top-up sessions over 1 week (Figure 1). For the two top-up sessions only, all participants received the normobaric hypoxic gas (i.e., both the previously hypoxic and normoxic-trained groups). This protocol was to test the effectiveness of a top-up dose on previously hypoxic-trained participants and to test whether a minimum dose of two hypoxic sessions had any beneficial effect on otherwise normally trained participants. Participants maintained their normal pre-season fitness and rugby training routines. As indicated previously, the repeat-sprint interval training programme was designed around GPS data from real match play (Jones M. R. et al., 2015) and consisted of 4 sets of 5 repetitions of 5 s all-out cycling efforts interspersed with 25 s active recovery low cadence cycling (~20–50 W) between efforts and 5 min active recovery low cadence cycling (~20–50 W) between sets. A 5 min warm-up at ~50 W interspersed with a 5 s sprint at the end of each minute was performed prior to the sprint training, making the total exercise time ~35 min per session and 280 min over the total study (i.e., a total of 280 min under hypoxia). All participants were given strong verbal encouragement to maintain effort throughout the training.

During training, subjects received either a normobaric hypoxic (HYP) or a normobaric normoxic gas (NORM) via the GO_2_Altitude® hypoxicator system (Biomedtech, Victoria, Australia). After calibrating the equipment at the start of each training session, the hypoxic or placebo (normoxic) gas was sent to two 100L Douglas bags connected in series. Participants breathed from the bags via a leak-free respiratory mask (Hans-Rudolph 8980, Kansas City, Missouri, USA) attached to a one-way non-rebreathing valve (Hans-Rudolph 2700). The fraction of inspired oxygen (F_I_O_2_) was set at 14.5% (~3000 m) for the HYP group, and 20.9% for the NORM. We selected this hypoxic level based on previous research which suggested an F_I_O_2_ of between 14.8 and 16.7% (2000–3000 m) increases the physiological stress during repeat-sprint training without exacerbating the speed decline during such training (Bowtell et al., 2014; Goods et al., 2014). Participants were unable to view any oxygen or blood saturation monitors during training and we are confident of the blinding procedure as when asked at the end of intervention one, only a small fraction (i.e., 2 out of 10 controls) thought they might be receiving a higher oxygen dose or not getting hypoxia at all.

Participants recorded their daily training information along with their subjective ratings of stress, fatigue, muscle soreness, quality of sleep, and quality of training performance. Previous research by our group (Hamlin and Hellemans, 2007) and other researchers (Eston and Williams, 1988) indicates that such effort ratings can be used as reliable indicators of exercise intensity. To compare the total training load between groups, training impulse (Trimp) (Banister and Calvert, 1980) was calculated, which was expressed as a product of stress (duration of training) and strain (subjective rating of training intensity). Participants reported their subjective rating with the use of the 15-point (6–20) Borg scale (Borg, 1982).

### Performance tests

Performance testing was composed of a warm-up, a squat jump (not reported here), a repeat sprint ability test, and a Yo-Yo Intermittent Recovery Level 1 test (YYIR1) followed by a warm-down. The order of testing was standardized and participants were rested for 10–15 min between each test. The warm-up consisted of a slow jog for 5 min followed by 5–10 min of dynamic and static stretching. The repeat sprint ability test consisted of eight maximal effort running sprints timed to go every 20 s. Times (to the nearest 0.01 s) for each sprint were recorded using two sets of electronic speed-timing lights (Smartspeed, Fusion Sport Ltd, Australia). Fatigue during the 8 sprints was calculated by two methods; (i) applying a straight line to the data and estimating the predicted time in the first minus the last sprint which was log-transformed to get percent fatigue and (ii) using the percentage decrement score as described by Glaister et al. (2008) (Fatigue = (100 × (total sprint time/ideal sprint time)) − 100). Finally, participants completed a 20-m shuttle run test (Yo-Yo Intermittent Recovery test Level 1, BangsboSport, Denmark, YYIR1). Testing was completed at the same time of day in a covered stadium on slip-free flooring under similar climatic conditions.

### Physiological measures

During the training sessions, heart rate was recorded (FT1; Polar, Kemple, Finland) and arterial oxygen saturation (SpO_2_, Sport-Stat; Nonin Medical, Minneapolis, Minnesota, USA) was monitored manually by the researchers, which was unable to be viewed by the participants. Participant's perceived exertion during sprint training (RPE) was recorded at the end of each set with the Borg scale (6–20).

### Statistical analysis

Changes in the mean of the variables and standard deviations representing the between-and within-subject variability were estimated using a mixed modeling procedure (Proc Mixed) in the Statistical Analysis System (Version 9.3, SAS Institute, Cary North Carolina, USA). We analyzed the natural logarithm of each measure to reduce any effects in non-uniformity of error and to obtain changes in measures and errors as percentages (Hopkins et al., 2009). The fixed effects were test time (the average of baseline 1 and 2, post 1, post 2 etc.), group (HYP, NORM) and their interaction. The random effects were subject and residual variance. Chances that the true effects were substantial were estimated with a spreadsheet (Hopkins, 2006), when a value for the smallest worthwhile effect was entered. We used a value of 1% for performance measures (Paton et al., 2001). For non-performance measures, we chose 0.20 standardized units (change in mean divided by the between-subject *SD* at baseline) as the smallest worthwhile change (Cohen, 1988). To make inferences about the true (population) values of the effect of hypoxia on performance, *P*-values, and statistical significance were not used. Instead, uncertainties in the estimate of changes were presented as 90% confidence intervals and as likelihoods that the true value of the effect was increased, decreased or trivial. The descriptors: increased, trivial or decreased were used to describe the direction of the change. Where the confidence interval spanned all three possibilities (increased, trivial and decreased), the result was deemed unclear. In all other cases, such as no overlap, or an overlap between two possibilities (trivial and increased, or trivial and decreased) a clear result was achieved. Finally, the magnitude or probability of the change was assessed using a qualitative scale defined as: <0.5%: almost certainly not; <5%: very unlikely; <25%: unlikely/probably not; 25–75%: possibly, possibly not; >75%: likely, probably; >95%: very likely; and >99.5%: almost certainly.

## Results

### Training variables

**Figure 2:**
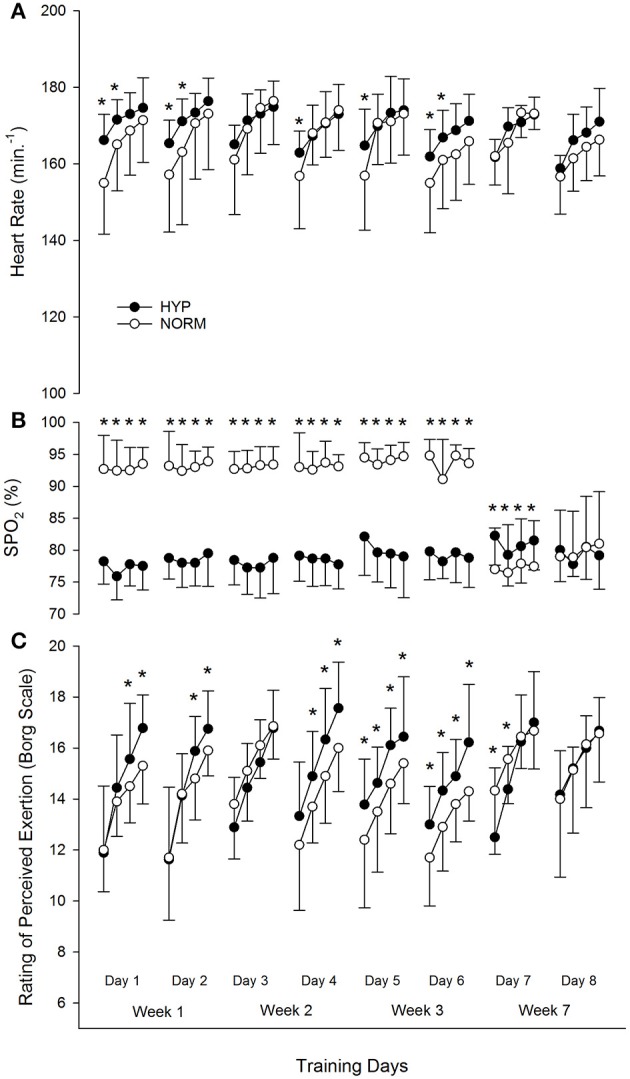
**Physiological and perceived exertion data**. ^*^Substantially different between groups at each time point. **(A)** Heart rate at the end of each set; **(B)** Arterial oxygen saturation at the end of each set; **(C)** Rating of perceived exertion (Borg 6–20 scale) at the end of each set.

We found no substantial difference in the training volume between groups measured as either training duration or training impulse (Trimp) per week (Table 1). The NORM group's mean SpO_2_ at the end of each sprint training set remained between 92 and 95% for the first 6 training days, whereas the HYP group's SpO_2_ was substantially lower at 77–82%. However, during the last 2 training days where all individuals (HYP and NORM groups) received the hypoxic gas, SpO_2_ levels were lower in the NORM group compared to the HYP group initially (training day 7) but was similar in both groups by training day 8 (Figure 2B). Relative to the NORM group, the HYP group's heart rate at the end of each sprint training set was consistently elevated, particularly in the first 2 sets throughout the training period, apart from the last 2 training days when both groups received the hypoxic gas during training where heart rates were similar. Ratings of perceived exertion tended to be higher after the last set of each training day. Overall, perceived exertion tended to be higher in participants undertaking repeat sprint training under hypoxic compared to normoxic conditions (Figure 2C). Similar to SpO_2_ and heart rate, the first day of hypoxic training for the NORM group (training day 7) increased their perceived exertion during training, compared to the HYP group. The average peak power measured in Watts produced during the sprint training (mean of the 20 repeat sprints for each day) was substantially higher in the NORM compared to the HYP group on days 4–6 (Table 2), however this difference disappeared once body weight was accounted for (i.e., W kg^−1^). The average peak power was maintained throughout the 8 training days despite the increased resistance on training days 3 and 4.

**Table 2:** **Peak power output for each training day**.

		**Intervention 1**						**Intervention 2**	
		**Day 1**	**Day 2**	**Day 3**	**Day 4**	**Day 5**	**Day 6**	**Day 7**	**Day 8**
Peak power (W)	HYP	822 ± 217	851 ± 194	821 ± 182	824 ± 181^*^	811 ± 209^*^	838 ± 196^*^	829 ± 220	906 ± 112
	NORM	985 ± 146	996 ± 183	953 ± 195	1012 ± 203	996 ± 203	1029 ± 207	911 ± 237	979 ± 163
Peak power (W kg^−1^)	HYP	10.2 ± 3.0	10.3 ± 3.0	10.2 ± 2.7	10.2 ± 2.5	9.98 ± 2.8	10.4 ± 3.1	10.4 ± 3.0	10.8 ± 2.8
	NORM	11.4 ± 2.3	11.4 ± 2.5	11.3 ± 2.1	11.6 ± 2.5	11.4 ± 2.6	11.8 ± 2.4	10.5 ± 2.9	11.0 ± 2.1

*Data are mean ± SD. HYP, hypoxic group; NORM, normoxic group; Peak power, the average peak power from the WattBike produced during the 20 all out 5-s sprints on each training day*.

* *Substantially different between groups*.

### Performance

**Figure 3:**
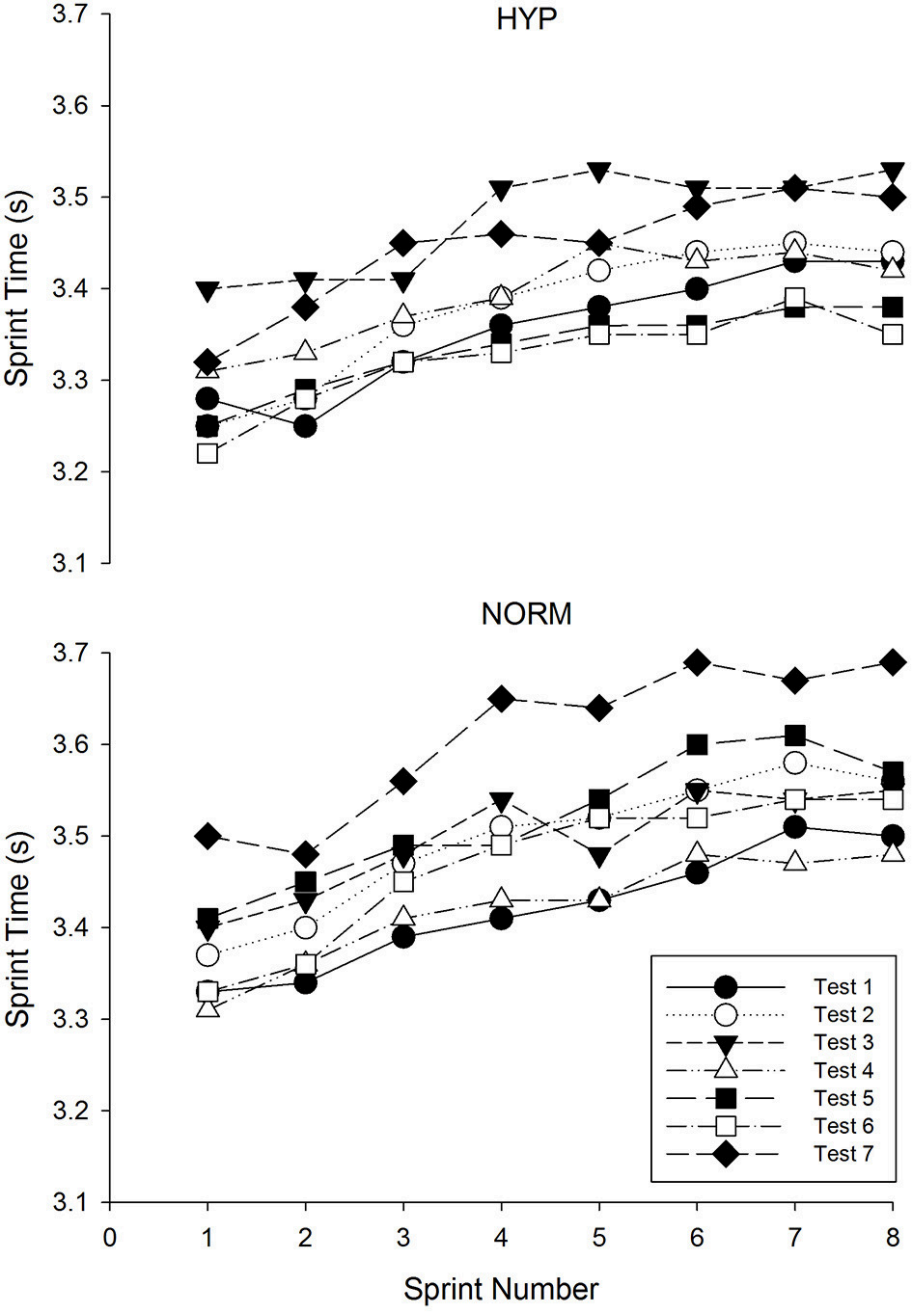
**Raw sprint times for the hypoxic (HYP) and normoxic (NORM) groups over the 7 testing periods**.

**Figure 4:**
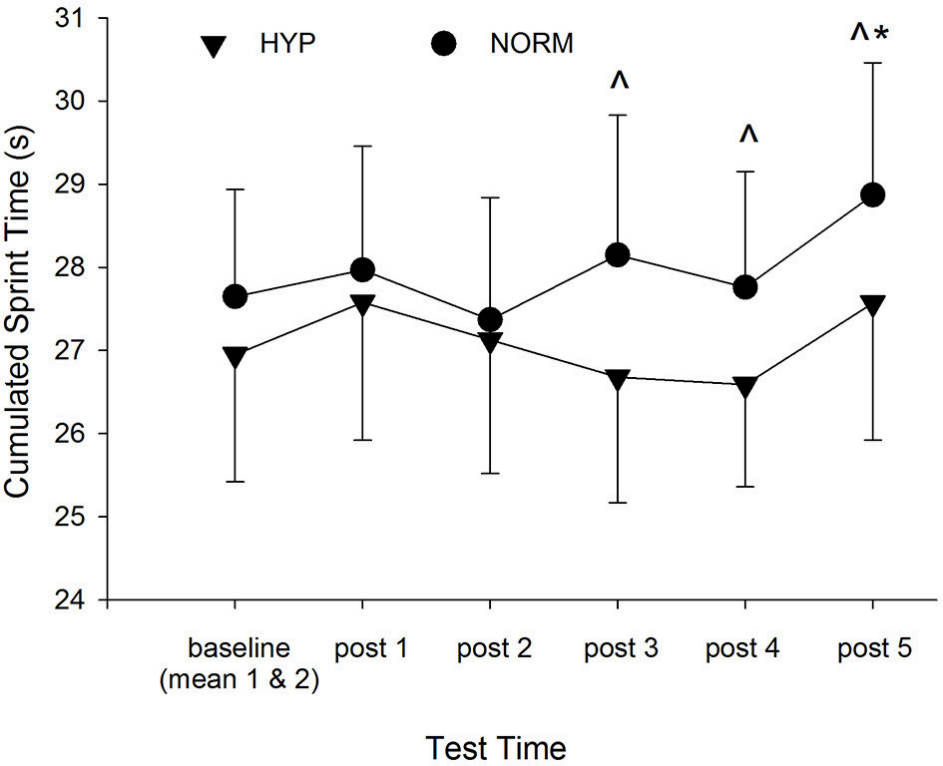
**Mean cumulated sprint time at baseline (mean of test 1 and 2) and the 5 post-intervention tests for the hypoxic (HYP) and normoxic (NORM) groups**. ^*^Substantially different from baseline. ^∧^Substantially different between groups at each time point.

Initially groups were matched for repeat sprint ability using the cumulated sprint time from test 1 (HYP 27.5 ± 3.9, NORM 27.4 ± 3.2) which was altered slightly due to the withdrawal of one participant's data from the hypoxic group (HYP 26.9 ± 3.4). Fatigue during the repeat sprint ability test was similar between groups at the two baseline tests when measured using either the linear extrapolation method (Baseline 1 test, HYP 5.8 ± 3.4%, NORM 5.5 ± 2.3% and Baseline 2 test, HYP 6.1 ± 2.6%, NORM 5.9 ± 1.8%, mean ± *SD*), or the percent decrement method (Baseline 1 test, HYP 3.5 ± 1.3%, NORM 3.5 ± 1.2% and Baseline 2 test, HYP 4.1 ± 1.1%, NORM 3.9 ± 1.4%). Using the linear extrapolation approach to measure fatigue, compared to the mean of the two baseline tests the HYP participants had substantially less fatigue during the repeated sprint tests on all post-test occasions (Table 3), however, this was only found in the NORM participants at post-test 1. Compared to the NORM participants, a beneficial effect of adding hypoxia to the repeat sprint training was indicated from post-test 3 onwards. Similar results were found using the percent decrement method to calculate fatigue (Table 3). Repeat sprint times for the 2 groups for each test day are presented in Figure 3. Test 1 had the lowest times for each sprint in the NORM group, whereas the lowest sprint times were witnessed on test day 5 and 6 (i.e., post-test intervention 3 and 4) for the HYP group. Cumulative times for the 8 sprints were similar between groups for the baseline and early post-intervention tests (post 1 and 2), however the HYP group improved cumulative sprint time compared to baseline at post-intervention 3 and 4 (1.0 ± 4.5 and 1.3 ± 4.5%, respectively, mean ± 90% CI, Figure 4). In comparison, over the same period the NORM group increased cumulative sprint time compared to baseline (1.7 ± 4.1 and 0.4 ± 4.4% for post-test 3 and 4, respectively). By post-intervention 5, both groups were slower over the 8 sprints compared to baseline (2.2 ± 4.2 and 4.3 ± 4.2% for the HYP and NORM groups, respectively). YYIR1 test performance improved throughout the study for both groups, with the effects of added hypoxia during repeat sprint training being unclear. Adding an extra two top-up sessions at the end of the 3-week post-intervention period had little beneficial effect in terms of further improving repeat sprint ability or YYIR1 performance in the HYP group over the next 2 weeks. Additionally, giving the NORM group two sessions of hypoxic training at the end of the study had little beneficial performance effect over the next 2 weeks (post 4 and 5). Standard deviations representing observed individual responses in performance in the post-exposure trials ranged from 1.7 to 2.8% for the repeat sprint ability test (using linear extrapolation) and 9.4–19.7% for the YYIR1 test. The typical error of the measurement for all participants between the two baseline tests was 0.8% (90% CL = 0.6–1.1%) and 4.7% (90% CL = 3.7–6.6%) for the repeat sprint ability and YYIR1, respectively.

**Table 3:** **Mean changes in performance tests post hypoxic and placebo exposures and the chances that the true differences in changes between groups is substantial**.

		**Change in mean (%)**			**Chances that the differences are substantial**	
		**Within-group, from baseline**		**Between-group**		
**Variable**	**Post-test**	**HYP ± 90% CL**	**NORM ± 90% CL**	**Difference ± 90% CL**	**%**	**Qualitative inference**
Repeated sprint fatigue^1^	1	−1.8 ± 1.6^*^	−1.5 ± 1.4^*^	0.3 ± 2.2	30	Unclear
	2	−2.1 ± 1.8^*^	−0.9 ± 1.5	1.2 ± 2.4	55	Unclear
	3	−2.3 ± 1.7^*^	−0.3 ± 1.7	2.0 ± 2.4^∧^	75	Possibly beneficial
	4	−1.9 ± 1.8^*^	0.4 ± 1.6	2.2 ± 2.4^∧^	81	Likely beneficial
	5	−1.2 ± 1.6^*^	0.5 ± 1.7	1.6 ± 2.4^∧^	67	Possibly beneficial
Repeated sprint fatigue^2^	1	−0.6 ± 0.9^*^	−0.1 ± 0.8	0.5 ± 1.2	27	Unclear
	2	−0.9 ± 0.9^*^	0.1 ± 0.8	1.0 ± 1.3^∧^	50	Possibly beneficial
	3	−1.2 ± 0.9^*^	−0.3 ± 0.9	0.9 ± 1.4^∧^	46	Possibly beneficial
	4	−0.6 ± 0.8^*^	0.4 ± 0.9	1.0 ± 1.3^∧^	53	Possibly beneficial
	5	−0.1 ± 0.9	1.1 ± 0.9^*^	1.2 ± 1.2^∧^	61	Possibly beneficial
Yo-Yo L1	1	15 ± 30	20 ± 26^*^	6 ± 39	58	Unclear
	2	23 ± 31	26 ± 26^*^	3 ± 38	53	Unclear
	3	26 ± 31^*^	13 ± 28	−13 ± 42	68	Unclear
	4	37 ± 31^*^	26 ± 28^*^	−11 ± 42	65	Unclear
	5	33 ± 29^*^	25 ± 31^*^	−9 ± 42	62	Unclear

* Substantially different from mean of baseline tests (see Table 1);

∧ *Substantially difference between groups. Repeated sprint fatigue^1^, % fatigue from sprint 1 to sprint 8 using the linear extrapolation method; Repeated sprint fatigue^2^, % fatigue decrement score using the % decrement score method (100 × (total sprint time/ideal sprint time) − 100); Yo-Yo L1 is % change in meters covered in the Yo-Yo intermittent recovery test Level 1*.

## Discussion

The novel findings of this study were that six sessions of repeat-sprint training under hypoxia, simulating rugby match conditions, while otherwise living at sea-level, had long-lasting beneficial effects on sea-level repeat sprint ability in field conditions. In addition, off-feet training (using a cycle ergometer) was beneficial at improving on-feet performance (run-based repeated sprinting) in rugby players. Finally, it seems that six sessions (2 per week for 3 weeks) of hypoxic repeat-sprint training is necessary for improvements in repeat sprint ability and two sessions over 1 week, had little effect at eliciting further enhancement in performance in previously hypoxic trained athletes or athletes new to hypoxic training.

The error of measurement in this study was similar to measures from previous studies ~1–2% for repeat sprints in team-sport athletes (Wood et al., 2006; Hamlin et al., 2012) and indicated good reliability of measures. Individual responses to hypoxia tended to be relatively small for repeat sprint ability and slightly larger for the YYIR1 test.

The hypoxic group in the present study had an ~2% greater improvement in their repeat sprint ability compared to the normoxic group over the last 3 testing trials (Table 3), which compares favorably to repeat-sprint training derived improvements in previous hypoxic studies (Jones B. et al., 2015; Brocherie et al., 2015b). In association with less fatigue in the HYP group, these players were also faster over the 8 sprints during this period compared to the NORM group and were ~1% faster at post-testing times three and four compared to baseline. However, not all researchers have found beneficial effects when adding hypoxia to repeat-sprint training (Galvin et al., 2013; Goods et al., 2015). We suggest the cause of some of the conflicting results are likely to be associated with the mismatch between hypoxic training protocols and subsequent performance testing procedures. For example, Galvin et al. (2013) had rugby players complete 12 sessions of ten 6 s all out sprints with a 30 s recovery on a non-motorized treadmill under hypoxic conditions, but used a performance test that did not match this protocol (i.e., 10 × 20 m running sprints with 30 s rest period). Data derived from the Galvin et al. (2013) study shows that the average time to run 20 m by these athletes was ~3 s. Therefore, to match the training program, these athletes should have been running ~40 m during performance testing. Such details are important, as during training, these athletes would have been stressing and subsequently adapting, more aerobic than anaerobic metabolic processes. But during testing, these athletes would have been relying to a greater extent on anaerobic rather than aerobic metabolic systems. Indeed, these authors reported substantial improvements in endurance (aerobic) performance after training (~15% improvement in hypoxic compared to normoxic group), which suggests the training was more conducive to endurance performance than anaerobic repetitive sprinting performance adaptations.

In contrast, Goods et al. (2015) used similar training and testing procedures to our study but found non-significant changes between normoxic and hypoxic groups post-exposure for repeat sprint running ability. These results conflict with the present study, which showed substantially improved repeat sprint running ability post hypoxic exposure. However, these researchers did not quantify the training loads between groups, which could possibly result in different training stress and therefore adaptation in the groups studied.

The different methods used to quantify the change in repeat sprint ability between studies may also influence results. A recent article suggests using the average or cumulated sprint time overcomes the problem of high variability associated with calculating fatigue scores from the first and last sprints in a set (Oliver, 2009). However, we have found that using a linear function to predict the first and last sprints of the log-transformed data, which can then be subtracted to give a percent fatigue index, can also produce meaningful fatigue values with a low co-efficient of variation (~1%). We acknowledge that using this approach may overestimate the final sprint in a set which can be slightly faster due to participants inadvertently pacing themselves (Glaister et al., 2008), however, we found substituting the predicted 8th sprint with the actual recorded 8th sprint had little effect on the results. Indeed, when we calculated fatigue using the method suggested by Glaister et al. (2008) which can be found in Table 3 under “Repeated sprint fatigue^2^,” we found similar results to the linear extrapolation method.

Performance response to repeat-sprint training under hypoxic conditions is variable and inconsistent. While this study and others (Faiss et al., 2015; Kasai et al., 2015; Brocherie et al., 2015a,b) have shown beneficial changes in repeat sprint ability after repeat-sprint training in hypoxia compared to normoxia, this positive result is not always found (Goods et al., 2015; Montero and Lundby, 2016). Methodological differences including participant characteristics and ability, motivation and encouragement during training and testing, selection, and timing of performance tests and time, degree and frequency of hypoxia may theoretically help explain this variability in performance change. More research investigating these variables may help reduce performance variation with such training.

Postulated mechanisms associated with improved repeat sprint ability after repeat-sprint training in hypoxia include augmented anaerobic glycolytic metabolism (Svedenhag et al., 1991), improved acid-base homeostasis (Nummela and Rusko, 2000) and increased muscle blood perfusion (Faiss et al., 2013b). The increased repeat sprint ability in the hypoxic compared to the normoxic group in this study along with no substantial between group change in the aerobic measure (YYIR1) suggests improvement in the anaerobic rather than the aerobic metabolism is involved. However, this remains speculative since no mechanistic variables were measured in this study.

Generally the addition of hypoxia during exercise results in substantially higher ratings of perceived exertion (Shephard et al., 1992; Buchheit et al., 2012; Goods et al., 2014). It seems that when exercising under hypoxic conditions, even when the workload is reduced to account for the lowered oxygen availability (Buchheit et al., 2012), participants perceive the exercise to be more difficult. Some of this increased effort is probably due to the increased ventilator drive required during hypoxic exercise (Katayama et al., 2001), but increased peripheral muscular sensation via accumulation of hypoxic metabolites (Hogan et al., 1999) is probably also involved. Exercise under hypoxic conditions can also have a negative effect on cerebral oxyhemoglobin levels (Monroe et al., 2016) which may affect sensations directly. The increased perceived effort reported by the athletes in this study when adding normobaric hypoxia to high-intensity exercise (average training RPE increased from 14.7 to 15.6 in the hypoxic group compared to 13.9–15.5, in the normoxic group over the eight training sessions) is similar to previous research (Aliverti et al., 2011), but is in contrast to a recent study (Brocherie et al., 2016). Brocherie et al. (2016) found a slow reduction in the perceived effort reported by elite field hockey athletes as they completed six sessions of repeat-sprint training (four sets of five reps of 5 s running sprints in FIO2 ~14.5%) over 2 weeks (average training RPE decreased from 14.6 to 13.1 in the hypoxic group and increased from 14.4 to 14.8 in the normoxic group over six training sessions). Differences between studies may be due to the exercise mode (cycling compared to running), the caliber of athletes (elite field hockey players compared to well-trained rugby players), or the fact that athletes performed the training while resided at a simulated altitude of ~3000 m (Brocherie et al., 2016). In addition, the current study design with an increased resistance during training days 3 and 4 and a 3-week break between intervention 1 and 2 probably did not allow for such acclimation to be studied as rigorously as Brocherie's study.

A limitation to the current study was the fact that when participants were exercising under hypoxia the training stress was substantially higher compared to participants completing the same exercise under normoxic conditions (as witnessed by the significantly higher training heart rates and perceived exertion, Figure 2). Therefore, we need to be careful when attributing the benefit of such training to hypoxia, since the performance benefits may simply be due to the harder training load in the hypoxic group. Designing a study that could control for training workload in the hypoxic and control groups would be one way to tease out this effect. A further limitation of this study is that we used well-trained rugby players in this study and therefore the results may not reflect what may occur in elite rugby players. Lastly this study was a field study rather than a lab-based study in order to improve the ecological value of the outcomes, however such studies also increase the risk of elevating variability due to extraneous variables. One such variable was the fact that all the rugby players in this study were required to play a pre-season game of rugby on the Saturday prior to the last testing day, which was probably responsible for the poor repeat sprint ability on the last testing day.

Finally, the present study found that “off-feet” repeat sprint cycling had a substantially beneficial effect on “on-feet” repeat sprint running ability in amateur rugby players. Such cross-over effects in performance enhancement is not always found in such studies (Goods et al., 2015), but indicates a potentially useful training protocol for athletic teams that have heavy on-feet training loads such as running. Currently the professional rugby season in New Zealand spans 9–10 months of the year (early February through to early November) which represents a large training load on players. Any opportunity to relieve on-feet training stress without reducing overall performance would be a useful supplementation to such athletes, however this research was conducted on amateur rugby players and would need to be replicated on professional players before firm recommendations for this group could be made.

## Author contributions

MH conceptualized and designed the study, MH, PO, HM, assisted in the planning and acquisition of data, MH, PO, HM, CL, CE helped with the analysis and interpretation of the data, critically revising the manuscript and adding important intellectual content. All authors gave approval for the final version of this manuscript to be published and agree to be accountable for all aspects of the work.

## Funding

The authors acknowledge the Lincoln University Research Fund (reference 2014-16E/INTF044) and Ara Institute of Canterbury for financial support for this project.

### Conflict of interest statement

The authors declare that the research was conducted in the absence of any commercial or financial relationships that could be construed as a potential conflict of interest.
